# *catena*-Poly[[[aqua­(pyridine-2,6-dicarboxyl­ato *N*-oxide-κ^2^ *O* ^1^,*O* ^2^)cobalt(II)]-μ-1,3-di-4-pyridylpropane-κ^2^ *N*:*N*′] dihydrate]

**DOI:** 10.1107/S1600536808032741

**Published:** 2008-10-18

**Authors:** Li-Jin Wang

**Affiliations:** a Department of Chemistry, Lishui University, Lishui 323000, Zhejiang, People’s Republic of China

## Abstract

In the title compound, {[Co(C_7_H_3_NO_5_)(C_13_H_14_N_2_)(H_2_O)]·2H_2_O}_*n*_, the Co^II^ atom is coordinated by two O atoms from a pyridine-2,6-dicarboxyl­ate *N*-oxide ligand, two N atoms from two 1,3-di-4-pyridylpropane ligands and one water mol­ecule, and displays a distorted square-pyramidal coordination geometry. The 1,3-di-4-pyridylpropane ligands link the Co^II^ atoms into an infinite zigzag chain parallel to [010]. The chains are further linked through O—H⋯O and C—H⋯O hydrogen bonds, forming a three-dimensional supra­molecular network.

## Related literature

For related literature on metal complexes with pyridine-2,6-dicarboxyl­ate *N*-oxide, see: Nathan *et al.* (1985 ▶); Wen *et al.* (2005 ▶, 2006 ▶); Wu *et al.* (2007 ▶). For related literature on metal complexes with 1,3-di-4-pyridylpropane, see: Konar *et al.* (2003 ▶); Lai & Tiekink (2004 ▶); Li *et al.* (2004 ▶).
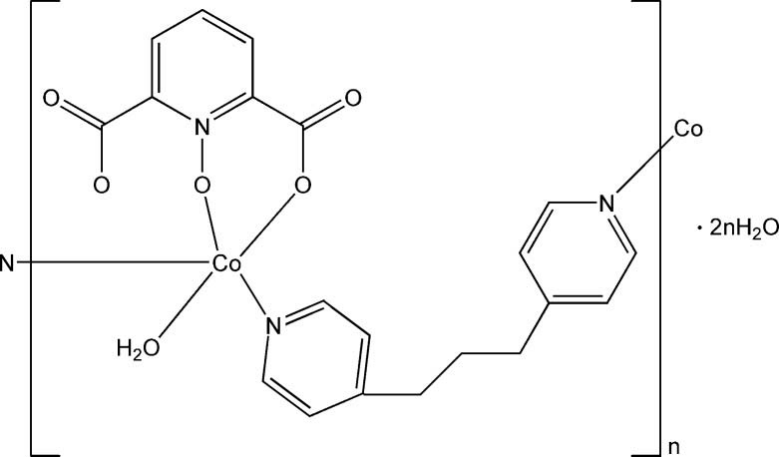


## Experimental

### 

#### Crystal data

[Co(C_7_H_3_NO_5_)(C_13_H_14_N_2_)(H_2_O)]·2H_2_O
*M* *_r_* = 492.34
Monoclinic, 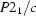

*a* = 10.2712 (12) Å
*b* = 11.5251 (13) Å
*c* = 18.309 (2) Å
β = 90.521 (2)°
*V* = 2167.3 (4) Å^3^
*Z* = 4
Mo *K*α radiation
μ = 0.84 mm^−1^
*T* = 296 (2) K
0.35 × 0.29 × 0.25 mm

#### Data collection

Bruker SMART APEXII CCD area-detector diffractometer
Absorption correction: multi-scan (*SADABS*; Sheldrick, 1996 ▶) *T*_min_ = 0.751, *T*_max_ = 0.817
10818 measured reflections
3897 independent reflections
2170 reflections with *I* > 2σ(*I*)
*R*_int_ = 0.064

#### Refinement

*R*[*F* ^2^ > 2σ(*F* ^2^)] = 0.059
*wR*(*F* ^2^) = 0.175
*S* = 1.00
3897 reflections
290 parameters
9 restraints
H-atom parameters constrained
Δρ_max_ = 0.66 e Å^−3^
Δρ_min_ = −0.40 e Å^−3^

### 

Data collection: *APEX2* (Bruker, 2007 ▶); cell refinement: *SAINT* (Bruker, 2007 ▶); data reduction: *SAINT*; program(s) used to solve structure: *SHELXS97* (Sheldrick, 2008 ▶); program(s) used to refine structure: *SHELXL97* (Sheldrick, 2008 ▶); molecular graphics: *SHELXTL* (Sheldrick, 2008 ▶); software used to prepare material for publication: *SHELXTL*.

## Supplementary Material

Crystal structure: contains datablocks I, global. DOI: 10.1107/S1600536808032741/hy2158sup1.cif

Structure factors: contains datablocks I. DOI: 10.1107/S1600536808032741/hy2158Isup2.hkl

Additional supplementary materials:  crystallographic information; 3D view; checkCIF report

## Figures and Tables

**Table 1:** Selected bond lengths (Å)

Co1—O1	1.927 (4)
Co1—O5	1.932 (3)
Co1—N2	1.994 (4)
Co1—N3^i^	2.000 (4)
Co1—O1*W*	2.184 (4)

Symmetry code: (i) 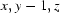.

**Table 2:** Hydrogen-bond geometry (Å, °)

*D*—H⋯*A*	*D*—H	H⋯*A*	*D*⋯*A*	*D*—H⋯*A*
O1*W*—H1*W*⋯O3^ii^	0.82	1.83	2.635 (5)	165
O1*W*—H2*W*⋯O2*W*	0.82	1.99	2.732 (6)	150
O2*W*—H3*W*⋯O3*W*^iii^	0.84	2.55	3.018 (9)	117
O2*W*—H4*W*⋯O3	0.84	1.98	2.778 (6)	157
O3*W*—H5*W*⋯O2	0.84	2.01	2.828 (7)	166
O3*W*—H6*W*⋯O4^iv^	0.82	2.19	2.935 (7)	150
C3—H3⋯O4^v^	0.93	2.46	3.364 (8)	165
C9—H9⋯O5^vi^	0.93	2.46	3.366 (7)	164
C15—H15*A*⋯O2^vii^	0.97	2.57	3.492 (7)	159
C18—H18⋯O3*W*^vi^	0.93	2.55	3.369 (7)	149

Symmetry codes: (ii) 
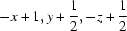
; (iii) 
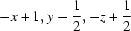
; (iv) 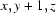; (v) 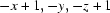; (vi) 
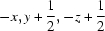
; (vii) 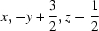.

**Fig. 1.**
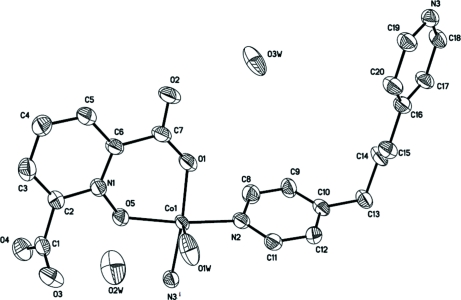
The structure of the title compound. Displacement ellipsoids are drawn at the 30% probability level. H atoms have been omitted for clarity. [Symmetry code: (i) x, -1 + y, z.]

**Fig. 2.**
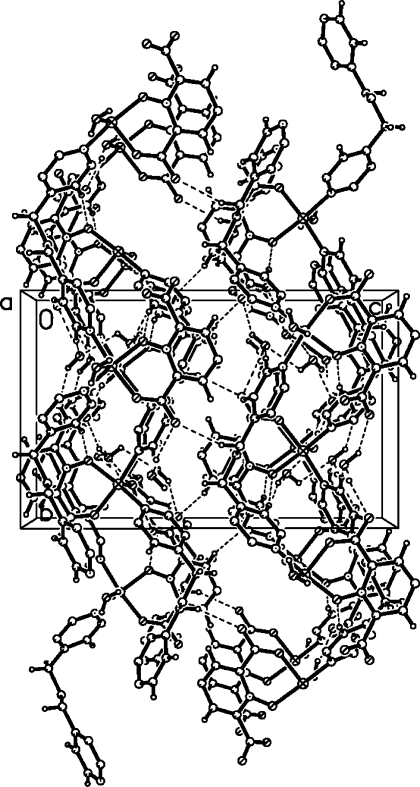
A packing view of the title compound. Hydrogen bonds are shown as dashed lines.

## supplementary crystallographic information

### Comment

In the structural investigation of metal complexes with pyridine-2,6-dicarboxylate-N-oxide (pdco), it has been found that pdco functions as a multidentate ligand with versatile coordination modes (Nathan *et al.*, 1985; Wen *et al.*, 2005, 2006; Wu *et al.*, 2007). As is well known, 1,3-di-4-pyridylpropane may act in bidentate bridging or monodentate terminal modes, leading to the formation of one-, two- or three-dimensional network (Konar *et al.*, 2003; Lai & Tiekink, 2004; Li *et al.*, 2004). On the basis of these observations, we utilize pdco, 1,3-di-4-pyridylpropane and Co^II^ ion as building blocks. A new one-dimensional coordination framework has been obtained from the hydrothermal treatment in an alkaline aqueous solution.

As illustrated in Fig. 1, the Co^II^ atom exists in a distorted square-pyramidal environment, defined by two O atoms from one pdco ligand, two N atoms from two 1,3-di-4-pyridylpropane ligands and one water molecule (Table 1). The O1, O5, N2, N3^i^ atoms (i = *x*, -1 + *y*, *z*) in the basal plane are alomst coplanar, and a water molecule lies at the apical position. The 1,3-di-4-pyridylpropane ligand in a bidentate bridging mode links the Co^II^ atoms into an infinite zigzag chain, with the shortest Co···Co separation of 11.525 (3)Å and a Co—C13—Co^ii^ angle (ii = *x*, 1 + *y*, *z*) of 100.06 (4)°. The chains are further self-assembled into a three-dimensional supramolecular network through O—H···O and C—H···O hydrogen bonds (Table 2; Fig. 2).

### Experimental

A mixture of cobalt chloride (0.238 g, 1 mmol), pyridine-2,6-dicarboxylic acid N-oxide (0.181 g, 1 mmol), 1,3-di-4-pyridylpropane (0.198 g, 1 mmol), NaOH (0.06 g, 1.5 mmol) and H_2_O (12 ml) was placed in a 23 ml Teflon-lined reactor, which was heated to 433 K for 3 d and then cooled to room temperature at a rate of 10 K h^-1^. The crystals obtained were washed with water and dryed in air.

### Refinement

H atoms bound to C atoms were positioned geometrically and refined as riding atoms, with C—H = 0.93(CH) and 0.97(CH_2_) Å and with *U*_iso_(H) = 1.2*U*_eq_(C). H atoms of water molecules were located on a difference Fourier map and fixed in the refinements, with *U*_iso_(H) = 1.5*U*_eq_(O).

### Crystal data

**Table:**

[Co(C_7_H_3_NO_5_)(C_13_H_14_N_2_)(H_2_O)]·2H_2_O	*F*(000) = 1020
*M**_r_* = 492.34	*D*_x_ = 1.509 Mg m^−^^3^
Monoclinic, *P*2_1_/*c*	Mo *K*α radiation, λ = 0.71073 Å
Hall symbol: -P 2ybc	Cell parameters from 5837 reflections
*a* = 10.2712 (12) Å	θ = 2.8–27.9°
*b* = 11.5251 (13) Å	µ = 0.84 mm^−^^1^
*c* = 18.309 (2) Å	*T* = 296 K
β = 90.521 (2)°	Block, colorless
*V* = 2167.3 (4) Å^3^	0.35 × 0.29 × 0.25 mm
*Z* = 4	

### Data collection

**Table:**

Bruker SMART APEXII CCD area-detector diffractometer	3897 independent reflections
Radiation source: fine-focus sealed tube	2170 reflections with *I* > 2σ(*I*)
graphite	*R*_int_ = 0.064
φ and ω scans	θ_max_ = 25.2°, θ_min_ = 2.0°
Absorption correction: multi-scan (SADABS; Sheldrick, 1996)	*h* = −12→12
*T*_min_ = 0.751, *T*_max_ = 0.817	*k* = −13→13
10818 measured reflections	*l* = −20→21

### Refinement

**Table:**

Refinement on *F*^2^	Secondary atom site location: difference Fourier map
Least-squares matrix: full	Hydrogen site location: inferred from neighbouring sites
*R*[*F*^2^ > 2σ(*F*^2^)] = 0.059	H-atom parameters constrained
*wR*(*F*^2^) = 0.175	*w* = 1/[σ^2^(*F*_o_^2^) + (0.0828*P*)^2^] where *P* = (*F*_o_^2^ + 2*F*_c_^2^)/3
*S* = 1.00	(Δ/σ)_max_ < 0.001
3897 reflections	Δρ_max_ = 0.66 e Å^−^^3^
290 parameters	Δρ_min_ = −0.40 e Å^−^^3^
9 restraints	Extinction correction: SHELXL97 (Sheldrick, 2008), Fc^*^=kFc[1+0.001xFc^2^λ^3^/sin(2θ)]^-1/4^
Primary atom site location: structure-invariant direct methods	Extinction coefficient: 0.0030 (8)

### Fractional atomic coordinates and isotropic or equivalent isotropic displacement parameters (Å^2^)

**Table:**

	*x*	*y*	*z*	*U*_iso_*/*U*_eq_	
O4	0.3287 (4)	−0.0221 (4)	0.4108 (2)	0.0776 (13)	
N3	0.2102 (4)	1.1756 (4)	0.1902 (2)	0.0519 (11)	
C19	0.2965 (6)	1.0894 (5)	0.1927 (3)	0.0622 (16)	
H19	0.3729	1.1009	0.2194	0.075*	
C20	0.2794 (6)	0.9841 (5)	0.1583 (3)	0.0609 (15)	
H20	0.3432	0.9272	0.1621	0.073*	
C18	0.1003 (5)	1.1549 (5)	0.1514 (3)	0.0568 (15)	
H18	0.0376	1.2129	0.1486	0.068*	
C17	0.0769 (6)	1.0515 (5)	0.1159 (3)	0.0597 (15)	
H17	−0.0007	1.0411	0.0902	0.072*	
C16	0.1677 (5)	0.9631 (5)	0.1180 (3)	0.0565 (14)	
C15	0.1470 (6)	0.8479 (5)	0.0794 (3)	0.0647 (16)	
H15A	0.1842	0.8532	0.0310	0.078*	
H15B	0.1948	0.7885	0.1059	0.078*	
Co1	0.24328 (6)	0.32149 (5)	0.24652 (3)	0.0423 (3)	
O5	0.2788 (4)	0.2215 (3)	0.32898 (19)	0.0565 (10)	
N2	0.1568 (4)	0.4237 (4)	0.1728 (2)	0.0522 (11)	
O2	0.2658 (5)	0.5550 (4)	0.4120 (2)	0.0892 (15)	
O1	0.2384 (4)	0.4531 (3)	0.3113 (2)	0.0777 (13)	
O3	0.4937 (4)	0.0354 (3)	0.3417 (2)	0.0761 (12)	
C6	0.3381 (5)	0.3644 (5)	0.4174 (3)	0.0513 (14)	
C12	0.1618 (6)	0.5381 (5)	0.0652 (3)	0.0593 (15)	
H12	0.2043	0.5575	0.0223	0.071*	
N1	0.3424 (4)	0.2545 (4)	0.3900 (2)	0.0494 (11)	
C3	0.4691 (6)	0.1910 (6)	0.4901 (3)	0.0702 (18)	
H3	0.5156	0.1326	0.5137	0.084*	
C7	0.2755 (6)	0.4653 (5)	0.3766 (3)	0.0607 (15)	
C2	0.4082 (5)	0.1683 (5)	0.4248 (3)	0.0496 (13)	
C13	−0.0066 (6)	0.6897 (4)	0.0347 (3)	0.0660 (17)	
H13A	−0.0981	0.6750	0.0252	0.079*	
H13B	0.0381	0.6903	−0.0117	0.079*	
C10	0.0487 (5)	0.5928 (5)	0.0824 (3)	0.0540 (14)	
C1	0.4076 (6)	0.0499 (5)	0.3885 (3)	0.0559 (14)	
C5	0.3971 (6)	0.3843 (5)	0.4831 (3)	0.0664 (16)	
H5	0.3931	0.4585	0.5029	0.080*	
C11	0.2145 (5)	0.4541 (5)	0.1107 (3)	0.0547 (14)	
H11	0.2918	0.4180	0.0976	0.066*	
C8	0.0451 (6)	0.4774 (5)	0.1890 (3)	0.0675 (17)	
H8	0.0037	0.4577	0.2322	0.081*	
C4	0.4619 (6)	0.2997 (6)	0.5211 (3)	0.0730 (18)	
H4	0.4999	0.3150	0.5663	0.088*	
C9	−0.0109 (6)	0.5589 (5)	0.1456 (3)	0.0653 (16)	
H9	−0.0899	0.5918	0.1587	0.078*	
C14	0.0088 (6)	0.8095 (4)	0.0717 (3)	0.0641 (17)	
H14A	−0.0387	0.8667	0.0432	0.077*	
H14B	−0.0299	0.8064	0.1198	0.077*	
O1W	0.4465 (4)	0.3311 (4)	0.2130 (3)	0.0969 (16)	
H1W	0.4767	0.3944	0.2011	0.145*	
H2W	0.4891	0.3034	0.2471	0.145*	
O2W	0.6417 (5)	0.2145 (5)	0.2840 (3)	0.1170 (19)	
H3W	0.6669	0.1724	0.2494	0.175*	
H4W	0.5887	0.1753	0.3084	0.175*	
O3W	0.2003 (5)	0.7719 (5)	0.3494 (4)	0.141 (2)	
H5W	0.2281	0.7061	0.3618	0.212*	
H6W	0.2190	0.8220	0.3795	0.212*	

### Atomic displacement parameters (Å^2^)

**Table:**

	*U*^11^	*U*^22^	*U*^33^	*U*^12^	*U*^13^	*U*^23^
O4	0.089 (3)	0.053 (3)	0.091 (3)	−0.012 (2)	0.005 (2)	0.013 (2)
N3	0.058 (3)	0.044 (3)	0.053 (3)	0.005 (2)	−0.012 (2)	0.000 (2)
C19	0.074 (4)	0.043 (4)	0.069 (4)	0.008 (3)	−0.015 (3)	−0.002 (3)
C20	0.066 (4)	0.045 (4)	0.071 (4)	0.008 (3)	−0.010 (3)	−0.005 (3)
C18	0.061 (4)	0.038 (3)	0.071 (4)	0.006 (3)	−0.012 (3)	0.002 (3)
C17	0.070 (4)	0.038 (3)	0.071 (4)	−0.008 (3)	−0.014 (3)	−0.004 (3)
C16	0.067 (4)	0.043 (3)	0.059 (3)	0.003 (3)	−0.002 (3)	−0.003 (3)
C15	0.080 (4)	0.045 (4)	0.069 (4)	−0.005 (3)	−0.003 (3)	−0.004 (3)
Co1	0.0543 (5)	0.0245 (4)	0.0478 (4)	0.0021 (3)	−0.0085 (3)	0.0006 (3)
O5	0.075 (3)	0.040 (2)	0.054 (2)	0.0000 (19)	−0.0150 (19)	−0.0013 (18)
N2	0.059 (3)	0.042 (3)	0.056 (3)	0.001 (2)	−0.002 (2)	−0.003 (2)
O2	0.133 (4)	0.046 (3)	0.088 (3)	0.019 (3)	−0.033 (3)	−0.024 (2)
O1	0.121 (4)	0.039 (2)	0.072 (3)	0.010 (2)	−0.026 (3)	−0.005 (2)
O3	0.074 (3)	0.048 (3)	0.107 (3)	0.005 (2)	0.022 (3)	−0.008 (2)
C6	0.055 (3)	0.040 (3)	0.059 (3)	0.007 (3)	−0.009 (3)	−0.008 (3)
C12	0.080 (4)	0.048 (4)	0.050 (3)	−0.005 (3)	−0.001 (3)	0.002 (3)
N1	0.055 (3)	0.040 (3)	0.053 (3)	0.001 (2)	−0.007 (2)	0.002 (2)
C3	0.076 (4)	0.066 (5)	0.068 (4)	0.010 (3)	−0.018 (3)	0.010 (3)
C7	0.071 (4)	0.045 (4)	0.066 (4)	0.009 (3)	−0.011 (3)	−0.013 (3)
C2	0.050 (3)	0.044 (3)	0.055 (3)	0.004 (3)	−0.001 (3)	0.004 (3)
C13	0.087 (4)	0.042 (3)	0.069 (4)	0.003 (3)	−0.027 (3)	0.003 (3)
C10	0.059 (3)	0.037 (3)	0.066 (4)	0.001 (3)	−0.013 (3)	−0.003 (3)
C1	0.064 (4)	0.041 (4)	0.062 (4)	0.007 (3)	−0.013 (3)	0.008 (3)
C5	0.086 (4)	0.047 (4)	0.066 (4)	0.006 (3)	−0.014 (3)	−0.008 (3)
C11	0.058 (3)	0.048 (4)	0.058 (3)	0.008 (3)	0.000 (3)	−0.003 (3)
C8	0.068 (4)	0.065 (4)	0.070 (4)	0.015 (3)	0.008 (3)	0.009 (3)
C4	0.088 (5)	0.065 (4)	0.066 (4)	−0.001 (4)	−0.029 (3)	−0.004 (3)
C9	0.068 (4)	0.056 (4)	0.072 (4)	0.015 (3)	0.006 (3)	0.012 (3)
C14	0.078 (4)	0.033 (3)	0.081 (4)	−0.004 (3)	−0.027 (3)	0.005 (3)
O1W	0.074 (3)	0.070 (3)	0.147 (4)	−0.008 (2)	−0.002 (3)	0.047 (3)
O2W	0.116 (4)	0.098 (4)	0.136 (5)	−0.009 (3)	−0.017 (3)	0.034 (4)
O3W	0.115 (4)	0.075 (4)	0.233 (7)	−0.015 (3)	−0.046 (4)	0.024 (4)

### Geometric parameters (Å, °)

**Table:**

O4—C1	1.232 (7)	C12—C10	1.361 (7)
N3—C19	1.332 (6)	C12—C11	1.384 (7)
N3—C18	1.348 (6)	C12—H12	0.9300
N3—Co1^i^	2.000 (4)	N1—C2	1.358 (6)
C19—C20	1.378 (7)	C3—C2	1.370 (7)
C19—H19	0.9300	C3—C4	1.377 (8)
C20—C16	1.379 (7)	C3—H3	0.9300
C20—H20	0.9300	C2—C1	1.518 (7)
C18—C17	1.378 (7)	C13—C10	1.524 (7)
C18—H18	0.9300	C13—C14	1.545 (7)
C17—C16	1.381 (7)	C13—H13A	0.9700
C17—H17	0.9300	C13—H13B	0.9700
C16—C15	1.519 (7)	C10—C9	1.371 (7)
C15—C14	1.492 (7)	C5—C4	1.368 (8)
C15—H15A	0.9700	C5—H5	0.9300
C15—H15B	0.9700	C11—H11	0.9300
Co1—O1	1.927 (4)	C8—C9	1.354 (7)
Co1—O5	1.932 (3)	C8—H8	0.9300
Co1—N2	1.994 (4)	C4—H4	0.9300
Co1—N3^ii^	2.000 (4)	C9—H9	0.9300
Co1—O1W	2.184 (4)	C14—H14A	0.9700
O5—N1	1.344 (5)	C14—H14B	0.9700
N2—C11	1.334 (6)	O1W—H1W	0.8200
N2—C8	1.339 (6)	O1W—H2W	0.8200
O2—C7	1.224 (6)	O2W—H3W	0.8400
O1—C7	1.259 (6)	O2W—H4W	0.8400
O3—C1	1.248 (7)	O3W—H6W	0.8200
C6—C5	1.361 (7)	O3W—H5W	0.8400
C6—N1	1.363 (6)	O3W—H6W	0.8200
C6—C7	1.522 (7)		
			
C19—N3—C18	116.1 (5)	C2—C3—C4	120.5 (6)
C19—N3—Co1^i^	119.9 (4)	C2—C3—H3	119.7
C18—N3—Co1^i^	123.9 (4)	C4—C3—H3	119.7
N3—C19—C20	123.9 (5)	O2—C7—O1	124.8 (6)
N3—C19—H19	118.0	O2—C7—C6	115.0 (5)
C20—C19—H19	118.0	O1—C7—C6	120.2 (5)
C16—C20—C19	120.1 (5)	N1—C2—C3	119.4 (5)
C16—C20—H20	119.9	N1—C2—C1	116.9 (4)
C19—C20—H20	119.9	C3—C2—C1	123.7 (5)
N3—C18—C17	122.9 (5)	C10—C13—C14	111.6 (4)
N3—C18—H18	118.5	C10—C13—H13A	109.3
C17—C18—H18	118.5	C14—C13—H13A	109.3
C18—C17—C16	120.6 (5)	C10—C13—H13B	109.3
C18—C17—H17	119.7	C14—C13—H13B	109.3
C16—C17—H17	119.7	H13A—C13—H13B	108.0
C20—C16—C17	116.3 (5)	C12—C10—C9	116.8 (5)
C20—C16—C15	121.0 (5)	C12—C10—C13	121.4 (5)
C17—C16—C15	122.7 (5)	C9—C10—C13	121.8 (5)
C14—C15—C16	115.6 (5)	O4—C1—O3	127.6 (6)
C14—C15—H15A	108	O4—C1—C2	117.4 (6)
C16—C15—H15A	108	O3—C1—C2	114.9 (5)
C14—C15—H15B	108	C6—C5—C4	122.7 (6)
C16—C15—H15B	108	C6—C5—H5	118.7
H15A—C15—H15B	107	C4—C5—H5	118.7
O1—Co1—O5	89.67 (16)	N2—C11—C12	121.5 (5)
O1—Co1—N2	86.43 (16)	N2—C11—H11	119.3
O5—Co1—N2	164.02 (17)	C12—C11—H11	119.3
O1—Co1—N3^ii^	166.97 (19)	N2—C8—C9	123.4 (6)
O5—Co1—N3^ii^	86.08 (16)	N2—C8—H8	118.3
N2—Co1—N3^ii^	94.30 (16)	C9—C8—H8	118.3
O1—Co1—O1W	99.39 (19)	C5—C4—C3	117.8 (5)
O5—Co1—O1W	94.32 (16)	C5—C4—H4	121.1
N2—Co1—O1W	101.61 (17)	C3—C4—H4	121.1
N3^ii^—Co1—O1W	93.21 (18)	C8—C9—C10	120.2 (6)
N1—O5—Co1	124.5 (3)	C8—C9—H9	119.9
C11—N2—C8	117.0 (5)	C10—C9—H9	119.9
C11—N2—Co1	122.3 (4)	C15—C14—C13	113.6 (5)
C8—N2—Co1	120.1 (4)	C15—C14—H14A	108.9
C7—O1—Co1	131.5 (4)	C13—C14—H14A	108.9
C5—C6—N1	117.8 (5)	C15—C14—H14B	108.9
C5—C6—C7	119.1 (5)	C13—C14—H14B	108.9
N1—C6—C7	123.0 (4)	H14A—C14—H14B	107.7
C10—C12—C11	121.0 (5)	Co1—O1W—H1W	118.8
C10—C12—H12	119.5	Co1—O1W—H2W	105.7
C11—C12—H12	119.5	H1W—O1W—H2W	110
O5—N1—C2	114.7 (4)	H3W—O2W—H4W	107
O5—N1—C6	123.5 (4)	H5W—O3W—H6W	112
C2—N1—C6	121.7 (4)		

Symmetry codes: (i) *x*, *y*+1, *z*; (ii) *x*, *y*−1, *z*.

### Hydrogen-bond geometry (Å, °)

**Table:**

*D*—H···*A*	*D*—H	H···*A*	*D*···*A*	*D*—H···*A*
O1W—H1W···O3^iii^	0.82	1.83	2.635 (5)	165
O1W—H2W···O2W	0.82	1.99	2.732 (6)	150
O2W—H3W···O3W^iv^	0.84	2.55	3.018 (9)	117
O2W—H4W···O3	0.84	1.98	2.778 (6)	157
O3W—H5W···O2	0.84	2.01	2.828 (7)	166
O3W—H6W···O4^i^	0.82	2.19	2.935 (7)	150
C3—H3···O4^v^	0.93	2.46	3.364 (8)	165
C9—H9···O5^vi^	0.93	2.46	3.366 (7)	164
C15—H15A···O2^vii^	0.97	2.57	3.492 (7)	159
C18—H18···O3W^vi^	0.93	2.55	3.369 (7)	149

Symmetry codes: (iii) −*x*+1, *y*+1/2, −*z*+1/2; (iv) −*x*+1, *y*−1/2, −*z*+1/2; (i) *x*, *y*+1, *z*; (v) −*x*+1, −*y*, −*z*+1; (vi) −*x*, *y*+1/2, −*z*+1/2; (vii) *x*, −*y*+3/2, *z*−1/2.

## References

[bb1] Bruker (2007). *APEX2* and *SAINT* Bruker AXS Inc., Madison, Wisconsin, USA.

[bb2] Konar, S., Zangrando, E., Drew, M. G. B., Mallah, T., Ribas, J. & Chaudhuri, N. R. (2003). *Inorg. Chem.* **42**, 5966–5973. 10.1021/ic034423o 12971767

[bb3] Lai, C. S. & Tiekink, E. R. T. (2004). *CrystEngComm*, **6**, 593–605.

[bb4] Li, X., Cao, R., Sun, D., Bi, W., Wang, Y., Li, X. & Hong, M. (2004). *Cryst. Growth Des.* **4**, 775–780.

[bb5] Nathan, L. C., Doyle, C. A., Mooring, A. M., Zapien, D. C., Larsen, S. K. & Pierpont, C. G. (1985). *Inorg. Chem.* **24**, 2763–2766.

[bb6] Sheldrick, G. M. (1996). *SADABS* University of Göttingen, Germany.

[bb7] Sheldrick, G. M. (2008). *Acta Cryst.* A**64**, 112–122. 10.1107/S0108767307043930 18156677

[bb8] Wen, L.-L., Dang, D.-B., Duan, C.-Y., Li, Y.-Z., Tian, Z.-F. & Meng, Q.-J. (2005). *Inorg. Chem.* **44**, 7161–7170. 10.1021/ic0509985 16180879

[bb9] Wen, L.-L., Tian, Z.-F., Lin, J.-G., Zhu, H.-Z. & Meng, Q.-J. (2006). *Z. Anorg. Allg. Chem.* **632**, 689–694.

[bb10] Wu, W.-P., Wang, Y.-Y., Wu, Y.-P., Liu, J.-Q., Zeng, X.-R., Shi, Q.-Z. & Peng, S.-M. (2007). *CrystEngComm*, **9**, 753–757.

